# Normative data and psychometric properties of the strengths and difficulties questionnaire among Japanese school-aged children

**DOI:** 10.1186/1753-2000-8-1

**Published:** 2014-01-21

**Authors:** Aiko Moriwaki, Yoko Kamio

**Affiliations:** 1 Department of Child and Adolescent Mental Health, National Institute of Mental Health, National Center of Neurology and Psychiatry, 4-1-1 Ogawa-Higashi, Kodaira, Tokyo 187-8553, Japan

**Keywords:** Child mental health, Questionnaire, Reliability, Validity, Normative banding, Strengths and difficulties questionnaire

## Abstract

**Background:**

Although child mental health problems are among the most important worldwide issues, development of culturally acceptable mental health services to serve the clinical needs of children and their families is especially lacking in regions outside Europe and North America. The Strengths and Difficulties Questionnaire (SDQ), which was developed in the United Kingdom and is now one of the most widely used measurement tools for screening child psychiatric symptoms, has been translated into Japanese, but culturally calibrated norms for Japanese schoolchildren have yet to be established. To this end, we examined the applicability of the Japanese versions of the parent and teacher SDQs by establishing norms and extending validation of its psychometric properties to a large nationwide sample, as well as to a smaller clinical sample.

**Methods:**

The Japanese versions of the SDQ were completed by parents and teachers of schoolchildren aged 7 to 15 years attending mainstream classes in primary or secondary schools in Japan. Data were analyzed to describe the population distribution and gender/age effects by informant, cut-off scores according to banding, factor structure, cross-scale correlations, and internal consistency for 24,519 parent ratings and 7,977 teacher ratings from a large nationwide sample. Inter-rater and test-retest reliabilities and convergent and divergent validities were confirmed for a smaller validation sample (total *n* = 128) consisting of a clinical sample with any mental disorder and community children without any diagnoses.

**Results:**

Means, standard deviations, and banding of normative data for this Japanese child population were obtained. Gender/age effects were significant for both parent and teacher ratings. The original five-factor structure was replicated, and strong cross-scale correlations and internal reliability were shown across all SDQ subscales for this population. Inter-rater agreement was satisfactory, test-retest reliability was excellent, and convergent and divergent validities were satisfactory for the validation sample, with some differences between informants.

**Conclusions:**

This study provides evidence that the Japanese version of the SDQ is a useful instrument for parents and teachers as well as for research purposes. Our findings also emphasize the importance of establishing culturally calibrated norms and boundaries for the instrument’s use.

## Background

Mental health problems affect 10-20% of children and adolescents worldwide [1], and substantial evidence indicates continuity in psychopathology from childhood into adulthood [2-4]. Despite heightened public concern in Japan for childhood mental health problems [5-7], many of these children remain unidentified and have no access to professional support due to various barriers including an insufficient specialized community health service system and parents or school teachers having inadequate knowledge of and stigma against child mental health problems. Recognizing this urgency, the Japanese Ministry of Health, Labour and Welfare has provided basic training opportunities for primary health professionals and promoted multidisciplinary work in the community since 2008. In addition, in 2009, the Ministry of Education, Culture, Sports, Science and Technology revised the School Health Act to strengthen the role that school personnel play in the early identification of children with mental health problems.

To support such initiatives, we need to develop reliable and valid measurement tools of psychopathological symptoms in Japanese children. At present, among the various questionnaires available for measuring mental health problems in children and adolescents, the Child Behavioral Checklist (CBCL) [8] has long been viewed as the “gold standard” because of its comprehensive nature. Although the CBCL is a solid instrument for conducting in-depth assessment, the 25-item Strengths and Difficulties Questionnaire (SDQ) [9] may be more suitable for screening purposes. The SDQ was created by Goodman by adding items on concentration, peer relations, and social competence to the established Rutter questionnaires. Because the SDQ measures not only behavioral problems but also the strengths of children and adolescents aged 4–16 years [10], parents and teachers can easily complete it. Furthermore, authorized translations of the SDQ are available free of charge [11]; http://www.sdqinfo.com. Due to its ease of use, the SDQ has now been translated into more than 75 languages and extensively validated in clinical and community samples [12-25]. These prior studies revealed that population-specific SDQ norms vary widely across countries.

To the best of our knowledge, only one study has examined the Japanese version of the SDQ. That study analyzed parent ratings in a community sample of 2,899 children aged 4–12 years [18] and found a gender effect on parent ratings, showed cut-off scores according to score banding, and confirmed its five-factor structure and satisfactory internal consistencies. However, given the value of having multiple informants reporting on children’s mental health problems especially for psychological assessment [26,27], we must examine whether its psychometric properties differ by rater. Also, to evaluate clinical usefulness, we need to examine it in a psychiatric clinical population as well as in a community population. The urgency to enhance school mental health care necessitates establishing culturally calibrated norms for Japanese schoolchildren based on a nationwide sample rather than on data from a restricted local area. Therefore, this study examined the applicability of the Japanese version of the SDQs for parents and teachers by establishing norms and cut-offs according to bandings and extending validation of its psychometric properties to a large, nationwide, and representative sample as well as a smaller clinical sample.

## Methods

This cross-sectional epidemiological study investigated the score distribution with gender and age effects, factor structure, reliability, and validity of the Japanese versions of the parent and teacher SDQs.

### Participants and data collection

Participants comprised a large-sized sample recruited from primary and secondary schools (normative sample) and a small-sized sample (validation sample) that was locally recruited. The schools were recruited countrywide with assistance from the Japanese Ministry of Education, Culture, Sports, Science, Technology and local government boards of education. We did not include private schools, national schools, or schools for handicapped children. Data were collected between December 2009 and March 2010 at the end of the Japanese school year to ensure that teachers knew their students well.

#### Normative sample

The parent SDQ to be completed at home was distributed to all parents of schoolchildren (aged 7–15 years) attending mainstream classes in 148 primary schools and 71 secondary schools in the 10 geographical areas making up Japan, with a letter from the investigators and school principals informing them about the study. From the parents of 87,548 children, 25,779 returned questionnaires to the investigators (29.4% response rate). Among these schools, 142 primary schools and 69 secondary schools (2,769 classes) agreed to participate in the teacher rating portion of the study. First, parents were informed about the study with a letter from the investigators and school principals. Second, among schoolchildren whose parents gave written consent, classroom teachers chose 4 children (2 boys, 2 girls) per class using a predetermined rule. In classes where less than 4 parents gave consent, teachers were asked to complete the questionnaire for all children whose parents who consented. We received 8,272 questionnaires rated by 2,183 teachers (78.8% response rate; 2,183/2,769). Among all questionnaires returned, we excluded 1,260 parent ratings (4.9%) and 295 teacher ratings (3.6%) with one or more missing answers, leaving 24,519 parent ratings (12,472 boys, 12,047 girls) and 7,977 teacher ratings (4,010 boys, 3,967 girls). Each of 9 grade levels comprised a minimum of 815 parent ratings and 302 teacher ratings for each gender (Table 1). The parent SDQ was rated by mothers (91.1%), fathers (7.6%), both parents (0.7%), and others (0.6%). The ratio of raters did not differ significantly between boys and girls (χ^2^ = 1.27, *ns*) or by age (χ^2^ = 2.11, *ns*). Therefore, the parent SDQ data rated by different raters were combined and analyzed in subsequent analyses.

**Table 1:** Number of children in the normative sample by gender and grade

**Grade**	**SDQ parent ratings (*n*** **= 24,519)**				**SDQ teacher ratings (*n*** **= 7,977)**			
	**Boys**	**%**	**Girls**	**%**	**Boys**	**%**	**Girls**	**%**
1	1,792	14.4	1,633	13.6	526	13.1	519	13.1
2	1,662	13.3	1,514	12.6	547	13.6	540	13.6
3	1,526	12.2	1,541	12.8	481	12.0	485	12.2
4	1,479	11.9	1,506	12.5	509	12.7	506	12.8
5	1,562	12.5	1,382	11.5	499	12.4	478	12.0
6	1,321	10.6	1,334	11.1	484	12.1	486	12.3
7	1,162	9.3	1,186	9.8	346	8.6	343	8.6
8	1,100	8.8	1,136	9.4	316	7.9	307	7.7
9	868	7.0	815	6.8	302	7.5	303	7.6
Total	12,472		12,047		4,010		3,967	

Note*. SDQ*, strengths and difficulties questionnaire. Most grade 1 participants were 7 years old at the time of the survey.

#### Validation sample

Participants were recruited from research volunteers with or without mental disorders, local schools, or a local pediatric outpatient clinic specializing in neurodevelopmental disorders. Participants totaled 128 children aged 6 to 16 years, of which 73 had any psychiatric diagnosis and 55 had no diagnosis (19 typically developing, 29 from community schools). Psychiatric diagnoses given by child psychiatrists or developmental pediatricians were autism spectrum disorder (*n* = 47), attention-deficit/hyperactivity disorder (*n* = 23), anxiety disorder (*n* = 2), specific phobia (*n* = 14), social phobia (*n* = 4), obsessive-compulsive disorder (*n* = 1), adjustment disorder (*n* = 2), tic disorders (*n* = 5), and others (*n* = 7). Thirteen of 73 children with any mental disorder had more than one diagnosis. Parent ratings were obtained for 108 children (69 clinical), and teacher ratings were obtained for 75 children (42 clinical). To examine inter-rater reliability, we used data from 63 participants rated by both parent and teacher at almost the same time. We collected retest data from the parents of 34 children 14 to 137 days later, and teachers of 18 children 10 to 107 days later (practical limitations precluded a shorter collection interval).

### Measures

#### Strengths and difficulties questionnaire

The SDQ is a 25-item questionnaire assessing child psychopathology and positive strengths of children and adolescents. Twenty-five items are classified into five subscales, four difficulties subscales (emotional symptoms, conduct problems, hyperactivity/inattention, peer problems) and one subscale on prosocial behavior. Each item is scored on a 3-point scale (0 = not true, 1 = somewhat true, 2 = certainly true). Each subscale score ranges from 0 to 10, and four difficulties subscale scores add up to a total difficulties score (range 0–40); higher difficulties scores indicate more difficulties, whereas the prosocial subscale score is reversely coded. The authorized Japanese translations of the SDQ [28] were used in this study.

#### Child behavioral checklist

The CBCL, a 113-item questionnaire assessing child psychopathology, comprises eight subscales (withdrawal problems, somatic complaints, anxious/depressed, social problems, thought problems, attention problems, delinquent behavior, aggressive behavior) [8]. After each item is scored on a 3-point scale, eight individual subscale scores, an internalizing score (withdrawal problems, somatic complaints, and anxious/depressed subscales), an externalizing score (delinquent and aggressive behavior subscales), and a total score can be calculated. The Japanese version was shown to be valid and reliable [29,30] and to have an 8-syndrome structure [31]. In this study, 46 parents and 29 teachers of primary schoolchildren in the validation sample completed the CBCL for Ages 4–18 (CBCL/4-18) and the Teacher Rating Form (TRF), respectively.

#### ADHD-rating scale-IV

The ADHD-Rating Scale-IV (ADHD-RS) is an 18-item questionnaire assessing symptom frequency characterized by attention deficit/hyperactivity disorder in children and adolescents [32]. Each item is scored on a 4-point scale, and inattention (sum of odd-numbered items), hyperactivity-impulsivity (sum of even-numbered items), and total score (sum of all items) can be calculated. The Japanese versions of the ADHD-RS home and school forms were shown to be valid, reliable, and to have a two-factor structure [33,34]. In this study, 41 parents and 43 teachers of primary schoolchildren completed the home form and school form, respectively.

### Ethical considerations

The study protocol was approved by the Ethics Committee of the National Center of Neurology and Psychiatry, Japan, and was performed in accordance with the ethical standards laid down in the 1964 Declaration of Helsinki and its later amendments. We obtained written informed consent to participate in this study from the caregivers of each child participant.

### Statistical analysis

Because the SDQ score distribution in the normative sample was significantly different from a normal distribution (Shapiro-Wilk and Kolmogorov-Smirnov tests, both *p* < .01), subsequent statistical analyses employed non-parametric tests. To examine gender effects, we used the Mann–Whitney U-test to compare scale scores between boys and girls. To examine age effects, we used the Kruskal-Wallis test and post-hoc Mann-Whitney’s comparisons with Bonferroni correction on the scale scores of three age groups (7–9, 10–12, 13–15 years). We conducted exploratory factor analysis (EFA) with varimax rotation and confirmatory factor analysis (CFA) on the normative sample to confirm the five-factor model. On the normative sample, we calculated internal consistency for the total difficulties score and each subscale score, and we assessed cross-scale correlations between the five scales using Spearman’s rank correlations. Inter-rater and test-retest reliabilities and convergent and divergent validities were assessed using Spearman’s rank correlations on the validation sample. We also examined temporal stability using a repeated-measures Wilcoxon signed-rank test on scores rated on two occasions for a smaller validation sample. All statistical analysis was performed with SPSS version 17.0 and AMOS version 10.0.

## Results

### Population distribution, and gender and age effects

Table 2 shows the means and standard deviations of parent- and teacher-rated SDQ scores in the normative sample, and also gender and age effects on the SDQ scores. Gender effects were significant for both parent and teacher ratings on total difficulties and all five subscale scores (total difficulties: *U* = 67,710,000, 5,796,000; emotional symptoms: *U* = 70,330,000, 7,782,000; conduct problems: *U* = 69,980,000, 6,558,000; hyperactivity/inattention: *U* = 61,150,000, 5,180,000; peer problems: *U* = 73,270,000, 7,140,000; prosocial behavior: *U* = 67,710,000, 5,796,000 [for parent and teacher ratings, respectively, *p* < 0.001 for all except teacher-rated emotional symptoms, *p* < 0.05 for teacher-rated emotional symptoms]). Parent ratings showed that boys scored significantly higher than girls on total difficulties and on the conduct problems, hyperactivity/inattention, and peer problems subscales, whereas girls scored significantly higher than boys on the emotional symptoms and prosocial behavior subscales. However, the effect sizes (*r*) of these gender differences were negligible. Teacher ratings, on the other hand, showed that boys scored significantly higher than girls on total difficulties and on all of the difficulties subscales, whereas girls scored significantly higher than boys on the prosocial behavior subscale. The effect sizes (*r*) of gender differences of teacher ratings on total difficulties and on hyperactivity/inattention and prosocial behavior subscale scores were small (0.24-0.31), although the rest were negligible (Table 2).

**Table 2:** Mean scores of parent- and teacher-rated SDQs and gender and age effects

	**Boys**		**Girls**		**Gender effect (*p, r *)**	**7-9 years**		**10-12 years**		**13-15 years**		**Age effect (*p, Cramer’s V *)**
**SDQ**	***M***	**(*SD *)**	***M***	**(*SD *)**		***M***	**(*SD *)**	***M***	**(*SD *)**	***M***	**(*SD *)**	
**Parent ratings**	(*n* = 12,472)		(*n* = 12,047)			(*n* = 9,968)		(*n* = 8,584)		(*n* = 6,267)		
Total difficulties	8.02	(5.26)	7.11	(4.76)	^‡^	8.39	(5.09)	7.20	(4.94)	6.82	(4.94)	^a‡ b‡ c‡, 0.15^
Emotional symptoms	1.31	(1.67)	1.49	(1.76)	^‡^	1.59	(1.77)	1.33	(1.67)	1.21	(1.68)	^a‡ b‡ c‡, 0.11^
Conduct problems	1.92	(1.59)	1.70	(1.43)	^‡^	2.01	(1.57)	1.74	(1.50)	1.62	(1.43)	^a‡ b‡ c‡, 0.12^
Hyperactivity/inattention	3.23	(2.30)	2.49	(1.98)	^‡^	3.27	(2.26)	2.69	(2.13)	2.49	(2.00)	^a‡ b‡ c‡, 0.16^
Peer problems	1.55	(1.69)	1.42	(1.50)	^‡^	1.52	(1.57)	1.44	(1.58)	1.51	(1.68)	^a‡^
Prosocial behavior	5.80	(2.15)	6.50	(2.08)	^‡^	6.18	(2.10)	6.26	(2.15)	5.91	(2.20)	^a† b‡ c‡^
**Teacher ratings**	(*n* = 4,010)		(*n* = 3,967)			(*n* = 3,098)		(*n* = 2,962)		(*n* = 1,917)		
Total difficulties	6.37	(5.80)	3.95	(4.50)	^‡^, 0.24	5.74	(5.70)	4.94	(5.22)	4.58	(4.79)	^a‡ c‡^
Emotional symptoms	0.82	(1.48)	0.77	(1.42)	^†^	0.93	(1.55)	0.76	(1.44)	0.64	(1.23)	^a‡ b‡ c†^
Conduct problems	1.20	(1.68)	0.68	(1.22)	^‡^	1.06	(1.61)	0.90	(1.45)	0.81	(1.35)	^a† c‡^
Hyperactivity/inattention	2.89	(2.67)	1.37	(1.76)	^‡^, 0.31	2.46	(2.60)	2.01	(2.32)	1.79	(2.04)	^a‡ c‡^
Peer problems	1.47	(1.86)	1.13	(1.56)	^‡^	1.30	(1.71)	1.28	(1.75)	1.34	(1.73)	
Prosocial behavior	5.73	(2.74)	7.14	(2.49)	^‡^, 0.26	6.47	(2.68)	6.48	(2.70)	6.28	(2.76)	^c†^

Note*. SDQ*, strengths and difficulties questionnaire. Age bands 7–9 years, 10–12 years, 13–15 years correspond to grades 1–3, 4–6, 7–9, respectively.

Age effect: ^a^7–9 yrs > 10–12 yrs, ^b^10–12 yrs > 13–15 yrs, ^c^7–9 yrs > 13–15 yrs. ^†^*p* < 0.05, ^‡^*p* < 0.001.

Age effects were also significant for both parent and teacher ratings except for the teacher-rated peer problem subscale. As for parent ratings, total difficulties and all subscale scores were significantly different by age band (total difficulties: *χ*^2^ = 568.33; emotional symptoms: *χ*^2^ = 307.30; conduct problems: *χ*^2^ = 323.96; hyperactivity/inattention: *χ*^2^ = 586.60; peer problems: *χ*^2^ = 19.26; prosocial behavior: *χ*^2^ = 88.62 [all *p* < 0.001]). Differences by age band were similar but diminished for teacher ratings (total difficulties: *χ*^2^ = 51.75; emotional symptoms: *χ*^2^ = 59.14; conduct problems: *χ*^2^ = 18.69; hyperactivity/inattention: *χ*^2^ = 71.61, all *p* < 0.001; peer problems: *χ*^2^ = 5.64, ns; prosocial behavior: *χ*^2^ = 6.77, *p* < 0.05). Post hoc comparisons between three age bands indicated that SDQ scores tended to be higher in younger children, as shown in Table 2. The effect size (Cramer’s *V*) of age effects was small for parent-rated total difficulties, emotional symptoms, conduct problems, and hyperactivity/inattention subscale scores, although negligible for all teacher-rated scores.

### Normative banding and cut-off score

Because gender or age effects were consistently observed for the total difficulties scores (Table 2), score ranges of the three bands (clinical, borderline, normal) were determined for the total difficulties scores by gender and age group (7–9, 10–12, 13–15 years) (Table 3). According to Goodman’s original work [10], the highest 10th percentile of the normative sample is defined as the “clinical” range, the next 10th percentile as the “borderline” range, and the remaining 80th percentile as the “normal” range. Although discrete scores made it impossible to divide the sample into exact percentiles, as Table 3 shows, nearly 10%, 10%, and 80% of the children were in the clinical, borderline, and normal bands.

**Table 3:** Normative banding of total difficulties score for parent- and teacher-rated SDQs for Japanese children

		**7-9 years**				**10-12 years**				**13-15 years**			
**SDQ**		**Boys**		**Girls**		**Boys**		**Girls**		**Boys**		**Girls**	
		** Raw score (%)**		**Raw score (%)**		**Raw score (%)**		**Raw score (%)**		**Raw score (%)**		**Raw score (%)**	
Parent rating	Normal	0-13	82.0%	0-11	81.0%	0-11	79.8%	0-10	82.0%	0-10	79.7%	0-10	81.5%
	Borderline	14-16	9.0%	12-14	9.7%	12-14	9.9%	11-13	8.2%	11-14	11.3%	11-13	8.9%
	Clinical	17-40	9.0%	15-40	9.3%	15-40	10.3%	14-40	9.8%	15-40	9.0%	14-40	9.6%
Teacher rating	Normal	0-11	78.9%	0-7	80.5%	0-10	78.1%	0-6	81.4%	0-9	81.3%	0-6	82.5%
	Borderline	12-16	11.6%	8-11	10.2%	11-14	10.8%	7-9	9.6%	10-12	8.9%	7-9	7.8%
	Clinical	17-40	9.5%	12-40	9.3%	15-40	11.1%	10-40	9.0%	13-40	9.8%	10-40	9.7%

Note*. SDQ*, strengths and difficulties questionnaire. There were no significant differences in proportion by age band between parent and teacher ratings for either boys or girls.

### Factor analysis

Table 4 shows rotated factor loadings for a five-factor EFA performed on parent- and teacher-rated SDQ scores with a rearranged item order. Only five factors had eigenvalues greater than 1.00, consistent with the original study [14] and the previous Japanese study [18]. EFA revealed that the five factors accounted for 33.03% and 55.22% of total variance of parent and teacher ratings, respectively, and most items loaded moderately to strongly onto their predicted factors. Communality values for teacher ratings were generally fair, at over 0.40 for 23 of 25 items, whereas only 7 of 25 items exceeded 0.40 for parent ratings. Parent- and teacher-rated item 7 (“obedient”) and teacher-rated item 14 (“popular”) loaded onto the prosocial factor more strongly than onto the predicted factor. The loading of parent-rated item 10 (“fidgety”) onto the emotional factor was also higher than that onto the predicted factor.

**Table 4:** Results of exploratory factor analysis (Varimax Rotation) of parent- and teacher-rated SDQs for Japanese children

**SDQ items**	**Parent ratings (*n*** **= 24,519)**						**Teacher ratings (*n*** **= 7,977)**					
	**Factor I**	**Factor II**	**Factor III**	**Factor IV**	**Factor V**	**Communality**	**Factor I**	**Factor II**	**Factor III**	**Factor IV**	**Factor V**	**Communality**
	***Pro ***	***Hyper***	***Emotion***	***Conduct***	***Peer***		***Pro***	***Hyper***	***Emotion***	***Conduct***	***Peer***	
Initial eigenvalue	4.88	2.60	1.70	1.21	1.12	11.52	7.07	2.60	1.82	1.24	1.08	13.80
% of variance	9.06	16.82	23.68	28.39	33.03		16.68	28.53	38.89	47.25	55.22	
**Prosocial behavior**												
1 considerate	-.63					.45	-.75					.65
4 shares	-.49					.26	-.64					.44
9 caring	-.66					.45	-.81					.69
17 kind to kids	-.53					.29	-.74					.57
20 helps out	-.58					.37	-.78					.63
**Hyperactivity/inattention**												
2 restless		.56				.46		.80				.74
10 fidgety		.27	.34			.27		.61				.56
15 distractive		.69				.63		.82				.77
21 reflective (*)		.56				.44		.57				.63
25 persistent (*)		.64				.50		.59				.59
**Emotional symptoms**												
3 somatic complaints			.31			.14			.54			.37
8 worries			.55			.37			.75			.59
13 unhappy			.44			.30			.65			.48
16 clingy			.62			.43			.68			.56
24 fears			.51			.29			.68			.51
**Conduct problems**												
5 temper				.45		.33				.57		.49
7 obedient (*)	.44			.28		.30	.54			.38		.44
12 fights				.46		.25				.67		.60
18 lies, cheats				.41		.31				.62		.54
22 steals				.23		.07				.58		.34
**Peer problems**												
6 solitary					.41	.21					.70	.55
11 good friend (*)					.38	.18					.61	.48
14 popular (*)					.42	.34	.55				.42	.59
19 picked on, bullied					.44	.33					.52	.44
23 best with adults					.50	.31					.68	.54

Note*. SDQ*, strengths and difficulties questionnaire. *indicates a reverse item and inverted scores were analyzed.

Furthermore, CFA results lend support to the five-factor structure of the SDQ; for the parent and teacher ratings, respectively, the comparative fit index was 0.83 and 0.86, the goodness of fit index was 0.93 and 0.89, the adjusted goodness of fit index was 0.91 and 0.86, and the root mean square error of approximation was 0.06 and 0.07. In addition, the 3 items (7, 10, 14) mentioned above were found to load onto the predicted factor with factor loadings >0.40 (0.43-0.75).

### Cross-scale correlations

Table 5 presents cross-scale correlations among five subscales by rater and gender. Correlations between externalizing-externalizing scales, that is, between conduct problems and hyperactivity/inattention, were strong (parent *ρ* = 0.48, teacher *ρ* = 0.53). By contrast, those between internalizing-externalizing scales were small (between emotional symptoms and conduct problems: parent *ρ* = 0.28, teacher *ρ* = 0.25; between emotional symptoms and hyperactivity/inattention: parent *ρ* = 0.28, teacher *ρ* = 0.32). Prosocial behavior was negatively correlated with externalizing behaviors (conduct problems, hyperactivity/inattention: parent *ρ* = 0.32, 0.31; teacher *ρ* = 0.50, 56, respectively) but showed little correlation with internalizing behaviors (emotional symptoms: parent *ρ* = −0.03, teacher *ρ* = −0.17). These findings were in line with the theoretical predictions, and common in boys and girls. All correlations were statistically significant at *p* < 0.01.

**Table 5:** Cross-scale correlations for parent- and teacher-rated SDQs of Japanese children aged 7–15 years (Spearman’s rho)

		**Parent rating (*n*** **= 24,519)**				**Teacher rating (*n*** **= 7,977)**			
**SDQ subscale**		**Conduct problems**	**Hyperactivity/inattention**	**Peer problems**	**Prosocial behavior**	**Conduct problems**	**Hyperactivity/inattention**	**Peer problems**	**Prosocial behavior**
Emotional symptoms	Boys	.29*	.31*	.33*	-.05*	.27*	.34*	.37*	-.18*
	Girls	.28*	.28*	.31*	-.04*	.23*	.33*	.37*	-.16*
	Total	.28*	.28*	.32*	-.03*	.25*	.32*	.37*	-.17*
Conduct problems	Boys		.50*	.24*	-.30*		.57*	.41*	-.50*
	Girls		.45*	.25*	-.33*		.45*	.41*	-.46*
	Total		.48*	.25*	-.32*		.53*	.42*	-.50*
Hyperactivity/inattention	Boys			.31*	-.28*			.41*	-.53*
	Girls			.28*	-.30*			.44*	-.52*
	Total			.30*	-.31*			.43*	-.56*
Peer problems	Boys				-.24*				-.46*
	Girls				-.25*				-.47*
	Total				-.24*				-.47*

Note*. SDQ*, strengths and difficulties questionnaire. Parent ratings: boys (*n* = 12,472), girls (*n* = 12,047). Teacher ratings: boys (*n* = 4,010), girls (*n* = 3,967).

*p < 0.01.

### Internal consistency

Table 6 shows that internal consistencies were generally good, with those of teacher ratings tending to be stronger than those of parent ratings. The relatively weak internal consistencies of conduct problems and peer problems might be explained by the cross-loadings of items 7 and 11 mentioned above. Cronbach’s α coefficients were very similar for boys and girls.

**Table 6:** Cronbach’s alpha coefficients for SDQ scores of Japanese children aged 7–15 years

**SDQ**	**Parent rating (*n*** **= 24,519)**			**Teacher rating (*n*** **= 7,977)**		
	**Boys**	**Girls**	**Total**	**Boys**	**Girls**	**Total**
Total difficulties score	.82	.79	.81	.86	.84	.86
Emotional symptoms	.64	.65	.64	.72	.72	.72
Conduct problems	.56	.50	.54	.69	.62	.67
Hyperactivity/inattention	.78	.73	.76	.85	.75	.84
Peer problems	.62	.54	.59	.70	.64	.68
Prosocial behavior	.72	.71	.73	.84	.82	.84

Note*. SDQ*, strengths and difficulties questionnaire.

### Inter-rater reliability

In a smaller subsample, parent-teacher correlations were found to be moderate for total difficulties scores (*n* = 63, 44 boys, 19 girls, mean age 9.0 ± 1.3 years, 42 with clinical diagnoses, 21 with no diagnoses; *ρ* = 0.40). Spearman’s rank correlation coefficients varied by subscale: emotional symptoms *ρ* = 0.49, conduct problems *ρ* = 0.33, hyperactivity/inattention *ρ* = 0.34, peer problems *ρ* = 0.50, and prosocial behavior *ρ* = 0.28. All were statistically significant (*p* < 0.01 for all scales except for prosocial behavior, *p* < 0.05 for prosocial behavior).

### Test-retest reliability

Thirty-four parents of a subsample (17 boys, 17 girls, mean age 10.4 ± 2.7 years, 19 with clinical diagnoses, 15 with no diagnoses) and 18 classroom teachers of children from community schools (12 boys, 6 girls, mean age 10.3 ± 2.8 years, 4 with clinical diagnoses, 14 with no diagnoses) completed the SDQ on two occasions (intervals: mean 54 ± 43 days, [14–137 days], mean 25 ± 25 days [10–107 days] for parents and teachers, respectively). Test-retest correlations of both parent and teacher ratings were excellent for total difficulties and all subscales (total difficulties *ρ* = 0.79, 0.95; emotional symptoms *ρ* = 0.80, 0.76; conduct problems *ρ* = 0.76, 0.88; hyperactivity/inattention *ρ* = 0.70, 0.84; peer problems *ρ* = 0.74, 0.79; prosocial behavior *ρ* = 0.87, 0.72; parent and teacher, respectively; all *p* < 0.01). Both parent and teacher ratings on two occasions did not significantly differ for any of the subscales except teacher-rated peer problems (*Z* = −2.14, *p* < 0.05, two-tailed test), indicating overall temporal stability.

### Convergent and divergent validity

Table 7 shows the correlations between parent-rated SDQ and CBCL/4-18 scores for 46 clinical patients (36 boys, 10 girls, mean age 8.0 ± 0.8 years) and those between teacher-rated SDQ and TRF scores for 29 clinical patients (23 boys, 6 girls, mean age 7.9 ± 0.7 years). SDQ total difficulties scores were strongly correlated with CBCL total scores for ratings by both parents and teachers (parent *ρ* = 0.56, teacher *ρ* = 0.77). Correlations between corresponding subscales of the SDQ and the CBCL were also moderate to strong: those between SDQ conduct problems scores and externalizing scores of the CBCL4-18/TRF (externalizing, delinquent behavior, aggressive behavior subscales) were strong (parent *ρ* = 0.50-0.66, teacher *ρ* = 0.66-0.80), whereas those between SDQ emotional symptoms scores and internalizing scores of the CBCL4-18/TRF (internalizing, withdrawal problems, somatic complaints, anxiety/depressed subscales) were moderate to strong (parent *ρ* = 0.40-0.52, teacher *ρ* = 0.50-0.57). All correlations were statistically significant (*p* < 0.01). By contrast, there were no significant correlations among subscales measuring conceptually different behaviors, as shown in Table 7.

**Table 7:** Correlations between the SDQ and CBCL for each rater (Spearman’s rho)

	**CBCL**	**Withdrawal problems**	**Somatic complaints**	**Anxiety/dep**	**Social problems**	**Thought problems**	**Attention problems**	**Delinquent behaviors**	**Aggressive behavior**	**Internalizing**	**Externalizing**	**Total**
	**SDQ**											
Parent rating (*n* = 46)	Total difficulties score	.32*	.44**	.25	.48**	.23	.62**	.54**	.43**	.36*	.51**	.56**
	Emotional symptoms	.40**	.48**	.44**	.23	.20	.19	-.03	.07	.52**	.05	.34*
	Conduct problems	.19	.21	.16	.06	.00	.37*	.66**	.50**	.21	.59**	.39**
	Hyperactivity/inattention	.06	.27	.00	.35*	.12	.58**	.49**	.39**	.09	.44**	.39**
	Peer problems	.20	.09	.05	.50**	.13	.32*	.11	.00	.10	.04	.18
	Prosocial behavior	-.26	-.15	-.03	-.07	-.06	-.27	-.30*	-.34*	-.16	-.37*	-.21
Teacher rating (*n* = 29)	Total difficulties score	.44*	.29	.49**	.75**	.48**	.82**	.55**	.68**	.48**	.68**	.77**
	Emotional symptoms	.23	.57**	.56**	.37*	.21	.33	.11	.18	.50**	.18	.36
	Conduct problems	.24	.05	.18	.60**	.33	.71**	.66**	.79**	.22	.80**	.66**
	Hyperactivity/inattention	.30	.22	.33	.52**	.36	.74**	.40*	.62**	.31	.59**	.66**
	Peer problems	.46*	-.05	.31	.75**	.55**	.66**	.51**	.53**	.33	.54**	.64**
	Prosocial behavior	-.34	-.03	-.15	-.43*	-.28	-.44*	-.14	-.46*	-.23	-.40*	-.40*

Note. *SDQ*, strengths and difficulties questionnaire. *CBCL*, child behavioral checklist. The subsample from which parent ratings were obtained (*n* = 46) consisted of clinical patients (36 boys, mean age 8.0 ± 0.8). The subsample from which teacher ratings were obtained (*n* = 29) consisted of clinical patients (23 boys, mean age 7.9 ± 0.7). *p < 0.05, **p < 0.01.

Similarly, Table 8 shows that SDQ hyperactivity/inattention subscale scores were strongly correlated with the ADHD-RS total scores as well as the inattention and hyperactivity/compulsion subscale scores for parent ratings (*n* = 41 from local schools, 25 boys, mean age 8.1 ± 1.5 years) and teacher ratings (*n* = 43 from local schools, 27 boys, mean age 8.1 ± 1.5 years). Strong correlations were also found between SDQ conduct problems subscale scores and ADHD-RS total and two subscales scores. By contrast, no significant correlation existed between the teacher-rated emotional symptoms subscale score and ADHD-RS score, although the correlation was moderate for the parent ratings.

**Table 8:** Correlations between the SDQ and ADHD-RS for each rater (Spearman’s rho)

**ADHD-RS**	**Parent rating (*n*** **= 41)**			**Teacher rating (*n*** **= 43)**		
**SDQ**	**Inattention**	**Hyperactivity/impulsivity**	**Total**	**Inattention**	**Hyperactivity/impulsivity**	**Total**
Total difficulties score	.76**	.67**	.77**	.73**	.65**	.74**
Emotional symptoms	.34*	.33*	.34*	.28	.17	.26
Conduct problems	.70**	.70**	.75**	.53**	.57**	.60**
Hyperactivity/inattention	.73**	.63**	.73**	.83**	.81**	.85**
Peer problems	.58**	.51**	.59**	.36*	.22	.33*
Prosocial behavior	-.29	-.26	-.31*	-.42**	-.86**	-.48**

Note. *SDQ*, strengths and difficulties questionnaire. ADHD-RS: ADHD-Rating Scale-IV. The subsample from which parent ratings were obtained (*n* = 41) consisted of primary schoolchildren (25 boys, mean age 8.1 ± 1.5). The subsample from which teacher ratings were obtained (*n* = 43) consisted of primary schoolchildren (27 boys, mean age 8.1 ± 1.5). *p < 0.05, **p < 0.01.

## Discussion

Our results provided normative data of parent and teacher SDQs for Japanese schoolchildren aged 7 to 15 years, and confirmed its reliability and validity.

### Gender and age effects in the general population

As for gender effects, both parents and teachers reported higher levels of difficulties for boys than for girls, except for emotional symptoms. Such gender differences in SDQ scores are well in line with previous SDQ studies across ages and countries [13,15-19,21-24] and in the original U.K. study [35]. In our study, observed gender differences were more pronounced in teacher ratings than parent ratings, a tendency that has also been reported in previous studies using SDQ [13,16,23,35,36]. A possible explanation for this tendency is that girls might be more able to adjust their behaviors to social situations than boys. Thus, we should exercise caution when interpreting information from parents and teachers when assessing clinical severity. Our finding of gender differences emphasizes the need to establish a culturally calibrated gender-specific norm for each SDQ rater version.

As for age effects, both parents and teachers reported the highest levels of difficulties for the youngest children, aged 7–9 years, although we found no systematic differences for either peer problems or prosocial behaviors. In our study, we found a robust line of descending tendency with age only for parent ratings; the effect size for teacher ratings was negligible. Many studies have reported a similar descending tendency of parent ratings with age [13,18,23,24,36], although no such age effect was found in community samples in Holland [19] or Hong Kong [16] or in an epidemiological sample in the United Kingdom [37]. By contrast, except for a study from Shanghai, China [13], almost all studies, including ours, found no systematic age difference for teacher ratings [16,23,36,38]. A Dutch study that examined parent, teacher, and self-ratings of the SDQ reported no age effect except in parent ratings [23]. Although ADHD prevalence decreases with development [39], a recent prospective and longitudinal study revealed that childhood-onset psychiatric disorders are relatively stable, and homotypic or heterotypic continuity is found for each disorder, especially behavioral disorders such as ADHD [37]. In other words, the descending tendency of parent ratings might reflect a phenotypic transition in their child rather than a true change in severity. Instead, as children get older, they might begin to conceal worries and problems from their parents. Therefore, researchers and clinicians might want to consider the clinical significance of gender and age differences when applying normative bandings to specific child populations [12].

Mean and cut-off scores of the Japanese version of the SDQ were lower than those for Europe, the United States, and China, although they were similar to those for Israel and Holland. These studies cannot be easily compared because the age ranges studied in their samples were not identical. However, the tendency for Japanese parents or teachers to give lower scores to children’s behaviors appears consistent among questionnaires such as the CBCL [29], ADHD-RS [33,34], and Social Responsiveness Scale [40,41]. One partial explanation for the relatively lower scores of Japanese children on behavioral measures such as the SDQ is that Japanese informants tend to respond to Likert-type ratings by choosing the scale’s midpoint, whereas U.S. informants tend to choose the scale’s extreme values [42]. In fact, if the original U.K. cut-off were applied to Japanese children, some Japanese children in the “clinical” range instead would be labeled “borderline”, and some labeled “borderline” would fall into the “normal” range. Thus, for both culturally appropriate use and cross-cultural research, we must establish national norms based on population distribution.

### Factor analysis

We confirmed the proposed five-factor structure for the Japanese version of the parent and teacher SDQs using EFA and CFA.

### Reliability and validity

Internal consistency, inter-rater reliability, and test-retest reliability of the Japanese version of the parent and teacher SDQs were generally satisfactory and comparable to the original version [14], and on the whole fell well within previously reported ranges [43]. On all subscales of internal consistency, teacher ratings were more reliable, a tendency that is in line with those of previous studies [43]. The test-retest interval of 10 days to 5 months in our study was wider than that in conventional measurement, but the test-retest reliability from our sample is comparable to that of samples with shorter intervals of 2 weeks to 2 months [13,16,19]. Therefore, the true test-retest reliability with a shorter interval might be even higher than the finding in the present study [14,15].

Regarding convergent validity, strong correlations between the SDQ and CBCL support that, overall, the Japanese SDQ measures the same construct that the Japanese CBCL measures, as shown in many studies [43]. Again, the correlation was higher for teacher ratings than for parent ratings. At the subscale level, correlations between SDQ behavioral difficulties subscales (e.g., conduct problems and hyperactivity/inattention subscales) and corresponding CBCL subscales were higher than the correlation between the SDQ emotional symptoms subscale and the corresponding CBCL subscale for both parent and teacher ratings. In addition, the SDQ hyperactivity/inattention subscale was highly correlated with the ADHD-RS measures for both parent and teacher ratings. This parent-teacher discrepancy or externalizing-internalizing discrepancy appears to be consistent with the studies reviewed by Stone [43].

### Limitations

This study has a number of limitations. First, despite a sufficiently large-sized normative sample, the validation sample was small and the clinical information was based on experts’ clinical judgment obtained without a validated structured interview in some cases. Thus, we could establish neither discriminant validity nor calculated sensitivity or specificity against psychiatric diagnoses. Second, the parent SDQ response rate was low (29.4%), although that of the teacher SDQ was acceptable (78.8%). Van Widenfelt et al. [23] pointed out that children of non-responding parents but not non-responding schools are likely to show higher scores. Also, we did not obtain demographic information (e.g., parental education level, income, and age; one- or two-parent family; number of siblings; teachers’ age and gender) that might be related to SDQ scores [12]. Therefore, the representativeness of our normative sample for parent ratings is unclear, although the normative sample rated by teachers was representative. Also, the influence of demographic factors on parents’ or teachers’ ratings is unclear. Third, because the age range of participants in the present study was restricted to school age (7–15 years), the applicability of the Japanese version of the SDQ for preschoolers is unknown. Fourth, we did not study the self-report version for adolescents aged approximately 11 to 16 years, who are an important target for community mental health service planning. Thus, a future study examining its usefulness as a screening tool must include detailed clinical data from a larger clinical sample and investigate its ability to discriminate between community and clinical samples and receiver operating characteristic curves. In addition, Japanese norms and psychometric properties of parent and teacher ratings for preschoolers and self-report for adolescents should be examined.

## Conclusions

This study provides gender- and age-specific norms by rater for Japanese schoolchildren and further evidence that the psychometric properties of the Japanese version of the parent and teacher SDQs are satisfactory. The findings indicate that the SDQ will serve as an efficient assessment tool of broad mental health problems in Japanese schoolchildren for research and clinical purposes, and that it is comparable to the original version and many other language versions. Our findings also emphasize the importance of establishing culturally calibrated norms and boundaries for each instrument’s use.

## Competing interests

The authors declare that they have no conflict of interest.

## Authors’ contributions

AM collected the data and performed the statistical analysis. YK designed the study and conducted the analysis. AM and YK wrote the manuscript. Both authors read and approved the final manuscript.
